# Antibacterial and Anti-Biofilm Properties of Diopside Powder Loaded with Lysostaphin

**DOI:** 10.3390/pathogens12020177

**Published:** 2023-01-23

**Authors:** Alina Kudinova, Alexander Grishin, Tatiana Grunina, Maria Poponova, Inna Bulygina, Maria Gromova, Rajan Choudhary, Fedor Senatov, Anna Karyagina

**Affiliations:** 1 Gamaleya National Research Center of Epidemiology and Microbiology, Ministry of Healthcare of the Russian Federation, 123098 Moscow, Russia; 2 All-Russia Research Institute of Agricultural Biotechnology, Russian Academy of Sciences, 127550 Moscow, Russia; 3 Center for Biomedical Engineering, National University of Science and Technology “MISIS”, 119049 Moscow, Russia; 4 Rudolfs Cimdins Riga Biomaterials Innovations and Development Centre of RTU, Institute of General Chemical Engineering, Faculty of Materials Science and Applied Chemistry, Riga Technical University, Pulka St 3, LV-1007 Riga, Latvia; 5 Baltic Biomaterials Centre of Excellence, Headquarters at Riga Technical University, Kipsala Street 6A, LV-1048 Riga, Latvia; 6 Belozersky Institute of Physico-Chemical Biology, Lomonosov Moscow State University, 119992 Moscow, Russia

**Keywords:** diopside, lysostaphin, ceramics, *Staphylococcus aureus*, implant, biofilm

## Abstract

Background: Diopside-based ceramic is a perspective biocompatible material with numerous potential applications in the field of bone prosthetics. Implantable devices and materials are often prone to colonization and biofilm formation by pathogens such as *Staphylococcus aureus*, which in the case of bone grafting leads to osteomyelitis, an infectious bone and bone marrow injury. To lower the risk of bacterial colonization, implanted materials can be impregnated with antimicrobials. In this work, we loaded the antibacterial enzyme lysostaphin on diopside powder and studied the antibacterial and antibiofilm properties of such material to probe the utility of this approach for diopside-based prosthetic materials. Methods: Diopside powder was synthesized by the solid-state method, lysostaphin was loaded on diopside by adsorption, the release of lysostaphin from diopside was monitored by ELISA, and antibacterial and anti-biofilm activity was assessed by standard microbiological procedures. Results and conclusions: Lysostaphin released from diopside powder showed high antibacterial activity against planktonic bacteria and effectively destroyed 24-h staphylococcal biofilms. Diopside-based materials possess a potential for the development of antibacterial bone grafting materials.

## 1. Introduction

Osteomyelitis is an infection of the bones and bone marrow that develops after trauma or surgery and is caused by bacteria, mainly *Staphylococcus aureus* [1,2]. The number of cases of osteomyelitis as well as the costs of medical treatment are very high worldwide. For example, in the U.S., where more than two million fixation devices are implanted annually, an infection during implantation develops in approximately 5% of cases, and treatment of each case costs, on average, more than 15,000 dollars [3]. Moreover, if standard procedure is followed, therapeutic failure or recurrence of infection occurs in 20% to 30% of cases [4,5], and the infection rates in elective surgery cannot be reduced below 1–2% [6]. A promising approach for the primary surgical treatment of fractures such as open fractures that may be complicated by infection, or the treatment of chronic osteomyelitis that requires the replacement of large areas of bone tissue, is the use of antibiotic-impregnated carriers [7,8,9,10]. Meanwhile, due to the ubiquity of MRSA strains of *S. aureus* [11], clinical treatment of osteomyelitis using standard antibiotics has become increasingly difficult. The effectiveness of antibiotic use is also negatively affected by the ability of *S. aureus* to form biofilms on medical implants [12], as biofilms make the bacteria significantly more resistant to antibiotics. One of the advanced ways to overcome these problems can be the use of enzymes that degrade bacterial cell wall components for impregnation in bone grafts instead of antibiotics. These enzymes are effective against both MRSA and MSSA strains of *S. aureus*.

In this work, we used peptidoglycan-degrading enzyme lysostaphin—a zinc-dependent peptidase that cleaves the pentaglycine cross-bridges in the peptidoglycan of *S. aureus*, which leads to the rupture of bacterial membrane and bacterial cell death [13]. Lysostaphin is highly active against both planktonic and biofilm forms of *S. aureus*, kills MRSA and MSSA strains with equal efficiency [14,15], and demonstrates synergy with conventional antibiotics [16], which can be beneficial if the use of lysostaphin-loaded materials is complemented by the standard antibiotic therapy. It has been successfully used to coat titanium plates used for bone fracture fixation [17] and has been incorporated into hydrogels injected into bone fractures [18,19], making it a suitable model protein for impregnating other bone graft materials as well.

Bioactive Ca-P ceramics, mainly hydroxyapatite (HA), the major mineral component of bone, can nonspecifically adsorb proteins and are often used as protein carriers in implants for bone grafting [20]. The calcium-magnesium silicate ceramic diopside (CaMgSi_2_O_6_), which is characterized by high bioactivity, biocompatibility, and better mechanical properties than HA, has recently been studied for use in regenerative medicine [21]. The high biocompatibility of diopside is attributed, in particular, to its ability of biomineralization [22]. In the first several hours after implantation or incubation in simulated body fluid, the surface of diopside is covered by a layer of apatite [23]. In addition, after implantation, diopside can release Ca^2+^, Mg^2+^, and SiO_3_^2−^ ions which enhance the proliferation of osteoblasts [24,25,26], providing osteoinductive properties to the carrier itself. These properties make diopside potentially promising as a replacement for calcium-phosphate ceramics such as HA for use as a protein carrier in implants. Recently, the adsorption capacity of diopside toward recombinant BMP-2 [27] has been shown to be three times greater than that of nano-HA [28]. Implants with diopside impregnated with BMP-2 demonstrated high osteoinductivity in vivo [28]. This suggests that diopside may be an effective carrier for other proteins as well.

The goal of the present study was to investigate the possibility of adsorption of lysostaphin on diopside powder and to evaluate the antibacterial and antibiofilm activity of the resulting material in vitro.

## 2. Materials and Methods

### 2.1. Production and Purification of Lysostaphin

Recombinant lysostaphin [29] was produced by autoinduction and purified by cation-exchange chromatography. The *E. coli* strain producing recombinant lysostaphin was inoculated into LB medium supplemented with 25 μg/mL kanamycin and 150 μg/mL ampicillin, incubated for 6 h at 37 °C and 110 rpm, diluted 1/1000 times with autoinduction medium (10 g/L tryptone, 5 g/L yeast extract, 25 mM NaCl, 25 mM KH_2_PO_4_, 5 mM (NH_4_)_2_SO_4_, 25 mM NH_4_Cl, 0.5% glycerol, 0.05% glucose, and 0.2% lactose), and cultivated for 16 h at 37 °C and 180 rpm. The biomass was collected by centrifugation and stored at −20 °C. To purify the recombinant protein, the biomass was thawed, suspended in the lysis buffer (0.1% Triton X-100, 100 mM NaCl, 1 mM PMSF, 1 mM MgCl_2_, 100 μg/mL lysozyme, 40 units/mL benzonaze, 20 mM Tris-HCl pH 8.0), and incubated at room temperature for 30 min. The lysate was further diluted 3 times with buffer A (50 mM NaCl, 20 mM Tris-HCl, pH 7.5), centrifuged at 10,000× *g* for 30 min, filtered through 0.4 μm syringe filter, and applied to a WorkBeads 40S column (Bio-works, Uppsala, Sweden) equilibrated with buffer A. The column was washed first with 0.1% Triton X-100, 50 mM NaCl, and 20 mM Tris-HCl, pH 7.5, then with 80 mM NaCl and 20 mM Tris-HCl, pH 7.5 and the recombinant protein was eluted with 180 mM NaCl and 20 mM Tris-HCl, pH 7.5. Eluted protein was dialyzed against water and treated with EDTA and ZnSO_4_ as described before [30] to ensure that the active site was occupied by Zn^2+^ ions. After treatment, lysostaphin was again dialyzed against water, aliquoted, and stored at −80 °C.

### 2.2. Diopside Synthesis and Characterization

Diopside (CaMgSi_2_O_6_) was synthesized by a ball-milling-assisted solid-state method as described earlier [28]. Briefly, an appropriate amount of powdered eggshells, synthetic magnesium oxide, and silica extracted from rice husk were mixed by using ball milling to reduce particle size as well as generate homogeneity. The precursor samples obtained after ball milling were transferred to a crucible and calcined at 1100 °C for 6 h. After calcination, the furnace was cooled to room temperature and the calcined product was grinded to fine powders prior to characterization. The resulting product was investigated using X-ray phase analysis (XRD, Difrey-401 (JSC Scientific Instruments, Saint-Petersburg, Russia), CrKα–radiation, λ = 2.29106 Å), Fourier transform infrared spectroscopy (FT-IR, Nicolet 380 spectrometer (Thermo Fisher Scientific, Waltham, MA, USA), 650–4000 cm^−1^), and scanning electron microscopy (SEM (VEGA3 TESCAN, Brno, Czech Republic), 20 kV).

### 2.3. Loading of Diopside with Lysostaphin

Diopside powder was incubated in 10 mM Tris-HCl, pH 7.5 for 10 min and centrifuged at 16,873× *g* for 10 min. The supernatant was removed and the protein solution was added to the diopside powder (100 μg protein per 10 mg diopside). The suspension was incubated at ambient temperature with constant shaking for 2 h. After that, the diopside was centrifuged at 16,873× *g* for 15 min, the supernatant was removed, and the diopside was washed with 10 mM Tris-HCl, pH 7.5. The concentration of protein in the supernatant and the wash buffer was measured using Bradford assay. The amount of adsorbed protein was calculated as the total protein minus the amount of unbound protein in the supernatant and the wash buffer.

### 2.4. Release of Lysostaphin from Diopside

Diopside powder loaded with lysostaphin was incubated in PBS with 1 mg/mL bovine serum albumin BSA, pH 7.4, at 37 °C with shaking. At 0, 1, 3, 5, 24, 48, and 72 h, the diopside powder was centrifuged, and the supernatant was withdrawn and replaced with the fresh PBS with 1 mg/mL BSA. The withdrawn supernatant was used to measure the concentrations (ELISA) and activity (drop plate assay) of released lysostaphin.

### 2.5. ELISA

The ELISA assay used to determine the concentrations of released lysostaphin was performed as described before [31,32]. Polyclonal rabbit antiserum raised against lysostaphin was diluted in 20 mM carbonate buffer, pH 9.7, and incubated overnight in a 96-well plate (Corning Inc., Corning, NY, USA) at +4 °C. The plate was washed twice with PBS with 0.05% Tween-20 (PBST), pH 7.4, and blocked with 1 mg/mL BSA in PBST for 2 h at +37 °C. The plate was again washed twice, the lysostaphin samples diluted in blocking buffer were added to the wells, and the plate was incubated overnight at +4 °C. After that, the plate was washed thrice with PBST, and anti-lysostaphin monoclonal antibodies (clone 2F9, State Research Center for Applied Microbiology and Biotechnology, Obolensk, Russia) in PBS were added to the wells. The plate was washed four times with PBST, and peroxidase-conjugated secondary antibodies (IMTEK, Moscow, Russia) in PBS were added to the wells. The plate was incubated for 1 h at +37 °C, washed five times with PBST, and filled with peroxidase substrate TMB (Life Technologies, Carlsbad, CA, USA). After the development of intensive color, the reaction was stopped by 10% sulfuric acid, and the optical density was measured at 450 nm using iMark plate reader (Bio-rad, Hercules, CA, USA).

### 2.6. Determination of Minimum Inhibitory Concentration (MIC) and Minimum Bactericidal Concentration (MBC)

To determine MIC of diopside loaded with lysostaphin, an overnight culture of *S. aureus* ATCC 29213 was grown on Tryptic Soy Broth without Dextrose medium (BD, Franklin Lakes, NJ, USA) with the addition of agar (1.5%) at 37 °C. The cells were suspended in saline to the turbidity of 0.5 McFarland, which is approximately 1.5 × 10^8^ cells/mL (DEN-1B McFarland Densitometer, Biosan, Riga, Latvia). The suspension was diluted to 5 × 10^5^ cells/mL with Mueller Hinton II Broth (BD) supplemented with 2% NaCl and 1 mg/mL BSA and placed into a 96-well cell culture microplate (Corning), 90 μL per well. After that, 10 μL of two-fold serial dilutions ranging from 0.5 to 0.0019 mg/mL of lysostaphin-loaded diopside (equivalent to approximately 2.6 to 0.01 μg/mL of loaded lysostaphin), 10 dilutions in total, were added. Each concentration was used in triplicate. The microplate was incubated on a thermo shaker (PST–60 HL plus, Biosan) at 270 rpm, 37 °C for 24 h. The presence/absence of visible *S. aureus* growth in the wells was determined, and the concentration of diopside resulting in the absence of growth was taken as MIC. Then, the content of each well of the microplate (a droplet of 3 µL) was plated on Petri dishes with Mueller Hinton II Broth (BD) with the addition of agar (1.5%) to determine the MBC. The growth of *S. aureus* on the Petri plates was observed the next day, and MBC was determined analogously to MIC.

### 2.7. Determination of Activity of Lysostaphin Released from Diopside

An overnight culture of *S. aureus* ATCC 29213 was grown on Tryptic Soy Broth without Dextrose medium (BD) with the addition of agar (1.5%) at 37 °C. The cells were suspended in saline to the 0.5 McFarland turbidity, and the suspension was evenly plated on Petri dishes with Mueller Hinton II Broth (BD) with the addition of agar (1.5%). The dishes were slightly dried until the liquid was completely absorbed into the agar. Samples containing the lysostaphin released from the diopside, obtained as described in 2.4, were dropped on the dishes in a volume of 10 µL at the initial concentration, diluted 2 times, and 10 times. The zones of absence of the bacterial growth were observed the next day.

### 2.8. Cultivation of Biofilms

An overnight culture of *S. aureus* ATCC 29213 was suspended in Tryptic Soy Broth (BD) supplemented with 2% NaCl, 1% glucose and 1.5% inactivated heparinized rat plasma to the concentration of 5 × 10^5^ cells/mL. Next, 800 µL of the resulting bacterial suspension was added to each well of a 24-well cell culture plate (SPL, Pocheon, Korea). Then, the microplate was incubated for 24 h without rocking at 37 °C on a thermo shaker (PST–60 HL plus, Biosan). To wash the biofilms, the remaining broth with planktonic cells was carefully dropped out, 800 µL of saline were added to each well, and the contents of the wells were again carefully dropped out. After washing, the biofilms were used for the determination of the anti-biofilm activity of the diopside.

### 2.9. Determination of Anti-Biofilm Activity of Diopside Loaded with Lysostaphin

To determine the anti-biofilm activity of control free lysostaphin, 800 µL of lysostaphin dilutions in PBS (pH 7.4) with 1 mg/mL BSA was added to grown biofilms of *S. aureus* ATCC 29213 cultivated at the bottom of the wells of 24-well plates (see 2.8). Diopside powder could not be directly added to the biofilms because it quickly settles down and can potentially physically disrupt the biofilms. To prevent this, the diopside loaded with lysostaphin was placed into special bucket-shaped inserts with a porous membrane bottom (SPLInsert 24-well, 3.0 µm pore size, SPL). These inserts were then placed into the plate wells, preventing direct contact of the insert content with the biofilms at the bottom of the well while still allowing the diffusion of released lysostaphin through the membrane. Each lysostaphin or diopside powder concentration was used in 3 replicates. Then, the 24-well microplates were incubated for 24 h at 37 °C with rocking at 50 rpm in an orbital shaker (OS-20, Biosan). 

To determine the impact of the treatment on the biofilm biomass, after incubation with diopside or lysostaphin, the biofilms were washed once with saline solution and dried at 60 °C in a thermo shaker (PST-60 HL plus, Biosan) for 1 h. Fixed biofilms were stained with 0.4% crystal violet for 15 min. The stained biofilms were washed in tap water and dried at room temperature. Then, 400 μL of 30% acetic acid were added to the wells of the microplate and incubated for 10 min, mixed, and 100 μL of solubilized stain was transferred into a clean 96-well microplate (Corning) (when necessary, the aliquot was diluted) and the optical density was determined at 550 nm on iMark microplate reader (Bio-rad).

To study the effect of the treatment on the viability of bacterial cells, the minimum bactericidal concentrations of planktonic (MBC-P) and biofilm-embedded (MBC-B) cell fractions were determined. To determine MBC-P, 10× serial dilutions of planktonic suspension from each well of the microplates were prepared, 5 µL of each dilution were transfer to the surface of a TSB agar plate (Tryptic Soy Broth medium (BD) with the addition of agar, 1.5%), and incubated overnight at 37 °C.

To determine MBC-B, after incubation, the biofilms were washed from planktonic cells and washed once with a saline solution. Then, 1 mL of saline solution was added to the biofilms and mixed by pipetting, 10× serial dilutions of bacterial suspension from each well of the microplate were prepared, and 5 µL of each dilution were transfer to the surface of a TSB agar plate and incubated overnight at 37 °C. 

MBC-P and MBC-P concentrations were determined by detecting the bacterial growth on the agar plates.

### 2.10. Statistical Analysis

An analysis of variance (ANOVA) was performed using R software version 4.2.2 [33]. The Dunnett test for comparing multiple treatments with a control was performed using the R package DescTools with a 95% family-wise confidence level.

## 3. Results

### 3.1. Characterization of Diopside Powder

**Figure 1:**
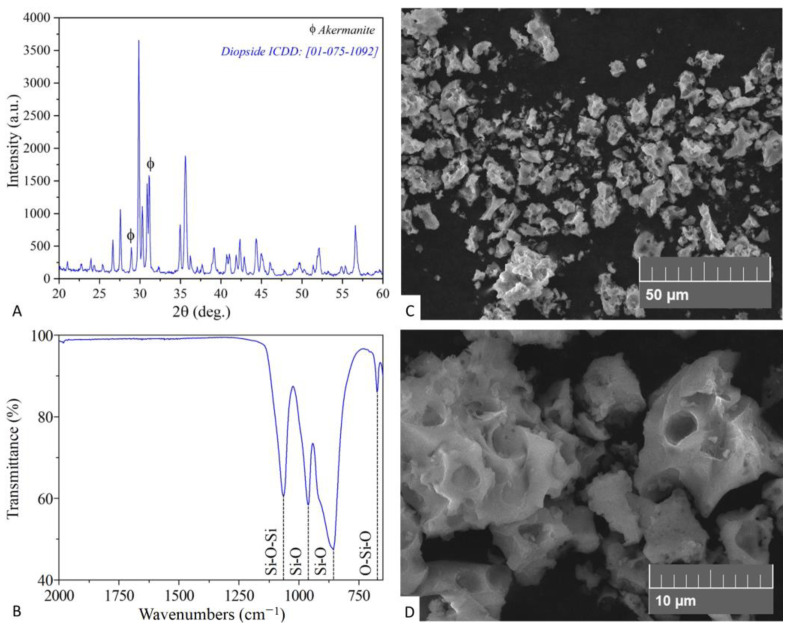
Properties of synthesized diopside powder. (**A**), XRD pattern; (**B**), FT-IR spectrum; (**C**,**D**), SEM images at different magnification.

Figure 1 depicts the characterization of synthesized bioceramic material. The data provided by XRD and FT-IR analysis demonstrated the formation of the diopside phase. The XRD pattern (Figure 1A) confirmed diopside formation as a primary phase. The observed phase matched the ICDD card № 01-075-1092. The FT-IR spectrum (Figure 1B) was found to be in good agreement with recently published diopside characterization results, demonstrating the presence of four functional groups’ peaks in the 650–4000 cm^−1^ range [34]. According to SEM micrographs (Figure 1C,D) the formed product is composed of aggregated porous microparticles.

### 3.2. Lysostaphin Adsorption to and Release from Diopside

Next, we determined the amount of lysostaphin that could be loaded onto the diopside powder. The diopside powder was incubated in the solution of lysostaphin and separated by centrifugation, and the concentration of unbound protein in the supernatant was determined. One mg of diopside powder adsorbed 5.20 μg of lysostaphin.

**Figure 2:**
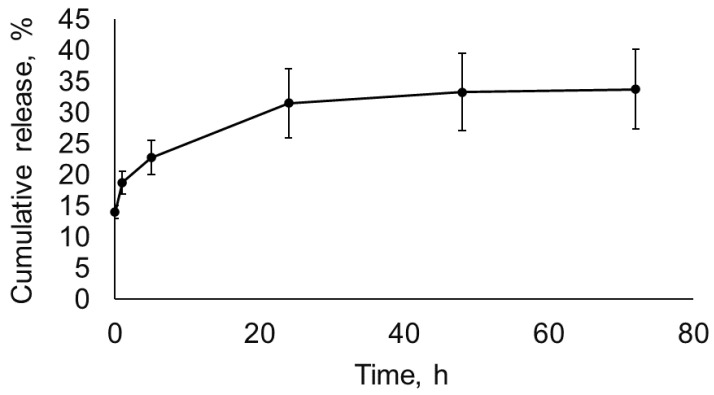
Cumulative release of lysostaphin from diopside powder measured by ELISA. Mean values of three repeats are shown, with error bars representing standard deviation.

**Figure 3:**
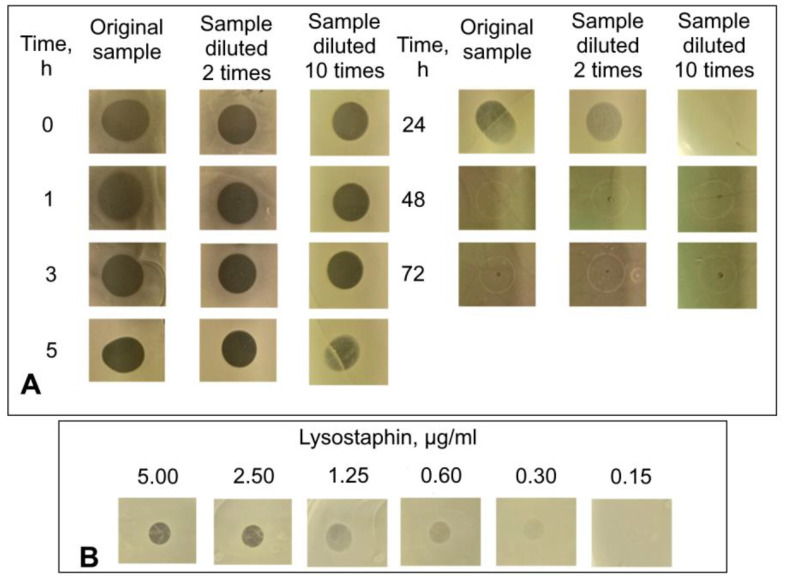
Antibacterial activity of lysostaphin released from the diopside powder. (**A**), the antibacterial activity of lysostaphin released from the diopside at different time points; (**B**), control activity of free lysostaphin. The dark round areas on agar plates are zones of inhibition of *S. aureus* growth.

To probe the kinetics of lysostaphin release from the diopside, the diopside powder was incubated in PBS for up to 72 h, and the concentration of protein released at different time points was determined by ELISA (Figure 2). In total, approximately 30% of the initially adsorbed lysostaphin was released from the diopside over the course of the experiment. Approximately two thirds of the released lysostaphin detached from the diopside during the first 5 h, and almost no lysostaphin was released after 24 h. To verify that the lysostaphin released from diopside retained its antibacterial activity, it was applied to a Petri plate inoculated with *S. aureus*, and the retardation of bacterial growth was evaluated. The minimal concentration of lysostaphin that could be detected in this assay was 0.30 μg/mL (Figure 3). The amounts of lysostaphin high enough to cause retardation of staphylococcal growth were released from the diopside for up to 24 h (Figure 3). Moreover, the samples of lysostaphin released at 0, 1, and 3 h could be diluted 10 times without affecting the zones of inhibition. Thus, the amount of lysostaphin released from diopside at these sampling points was no less than 3.00–6.00 μg/mL. This is in good accordance with the ELISA results. Thus, lysostaphin can be adsorbed to and released from diopside without a dramatic loss of activity.

### 3.3. Antibacterial and Anti-Biofilm Activity of Diopside Loaded with Lysostaphin

The antibacterial properties of diopside loaded with lysostaphin were estimated by its MIC and MBC values. The MIC of the diopside loaded with lysostaphin was 0.06 mg/mL, which is equivalent to approximately 0.31 μg/mL of adsorbed lysostaphin. The MBC of the lysostaphin-loaded diopside was 0.13 mg/mL, which corresponds to approximately 0.68 µg/mL of adsorbed lysostaphin. Control diopside powder did not demonstrate any antibacterial activity in our assay, while both the MIC and MBC of the free lysostaphin on the same *S. aureus* strain was 0.10 μg/mL [31,32]. Thus, diopside loaded with lysostaphin possessed significant antibacterial activity, which was, however, somewhat lower than the activity of an equivalent amount of free lysostaphin, corroborating the results of absorption and release experiments.

**Figure 4:**
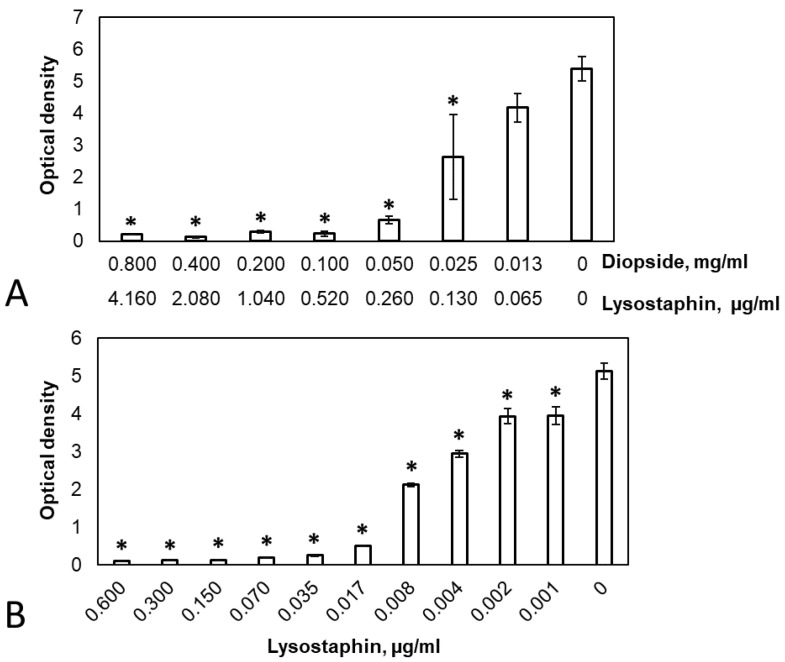
The anti-biofilm activity of diopside loaded with lysostaphin. (**A**), the crystal violet staining of the biofilms after treatment with different concentrations of the diopside loaded with lysostaphin (the concentrations of diopside and the approximate concentration of adsorbed lysostaphin are shown); (**B**), the crystal violet staining of the biofilms after treatment with different amounts of free lysostaphin. The optical density values that differ significantly from the control are marked with “*”, *p* < 0.05 (Dunnett’s test for comparing multiple treatments with a control). Mean values of three repeats are shown, with error bars representing standard deviation.

To determine the anti-biofilm properties of the diopside, pre-formed *S. aureus* biofilms were incubated in the presence of different concentrations of diopside loaded with lysostaphin for 24 h, and the amount of biofilm biomass remaining after the incubation was quantified by staining with crystal violet. Free lysostaphin was used for comparison. Both free lysostaphin (One way ANOVA: F(10, 22) = 417.8, *p* = 2 × 10^–16^) and diopside loaded with lysostaphin (One way ANOVA: F(7, 16) = 49.51, *p* = 1.16 × 10^–9^) demonstrated dose-dependent anti-biofilm activity, with a concentration of 0.02 μg/mL of free lysostaphin or 0.10 mg/mL of the diopside (equivalent to approximately 0.52 μg/mL of adsorbed lysostaphin) resulting in almost complete biofilm elimination (Figure 4). Control untreated diopside did not show any anti-biofilm properties.

We also determined the effect of diopside loaded with lysostaphin on the reduction of the CFU of *S. aureus* both in the planktonic and the biofilm cell fractions. The MBC of planktonic cells (MBC-P) or biofilm-embedded cells (MBC-B) were determined as the antibiotic concentration that reduced the number of CFU in a 24-h culture by at least three orders of magnitude [35]. Both the MBC-P and MBC-B of free lysostaphin were 0.07 µg/mL. The MBC-P of the lysostaphin-loaded diopside was 0.20 mg/mL (approximately 1.04 µg/mL of adsorbed lysostaphin), while the MBC-B was 0.10 mg/mL (approximately 0.52 µg/mL of adsorbed lysostaphin). As with the MIC values, MBC-P and MBC-B were higher for lysostaphin adsorbed on diopside compared to free lysostaphin. Interestingly, the different measures of anti-biofilm activity of the diopside (MBC-B for viable cells and crystal violet staining of total biofilm biomass) were in good accord with each other. The antibacterial (MIC) and antibiofilm (MBC-P, MBC-B, crystal violet staining) properties of diopside powder loaded with lysostaphin in comparison to free lysostaphin are summarized in Table 1.

## 4. Discussion

Diopside is a promising material with potential applications in regenerative medicine. It demonstrates good biocompatibility, mechanical properties, and stimulates the proliferation of osteoblasts. To further improve the properties and extend the range of applications of an implanted material, it is desirable to be able to functionalize it with additional active molecules such as various recombinant proteins. We have previously demonstrated that the loading of diopside-based material with recombinant BMP-2 enhances its osteoinductive properties [28]. In this work, we have addressed the possibility of loading diopside powder with antibacterial protein to lower the risks of implant-associated infections.

The amount of lysostaphin adsorbed to and released from the diopside powder was high enough to fully eliminate planktonic cells and eradicate biofilms of *S. aureus*. However, lysostaphin adsorbed less efficiently than previously reported recombinant BMP-2, with more than 150 μg of BMP-2 adsorbed per 1 mg of diopside [28] compared to only 5.20 μg of lysostaphin. This difference could be attributed to the differences in the general physicochemical properties of these proteins, such as solubility and isoelectric point. Another contributing factor could be the pH of the loading buffer. Lysostaphin was loaded onto the diopside powder at pH 7.5, while a buffer with pH 5.5 was used for BMP-2 due to its low solubility at a neutral pH. Although both proteins carry the same theoretically calculated total charge of approximately +10 at the respective pH values, the difference in pH might affect the properties of the diopside surface, leading to different adsorption capacity. If this is the case, the optimization of the loading protocol or the use of a different loading technique could potentially improve the diopside capacity for lysostaphin. Alternatively, specific interactions between proteins and certain sites on the diopside surface might be responsible for the differences in the adsorption capacities. In this situation, the fusion of lysostaphin with additional peptides capable of specific interactions with diopside could improve the loading efficiency and possibly the release kinetics.

Lysostaphin was continuously released from diopside powder over the course of 24 h, with the highest amount of lysostaphin released during the first 5 h and negligible amounts detected in the solution after 24 h. Interestingly, this release pattern is very similar to glycopeptide antibiotic vancomycin [36] and glucocorticoid dexamethasone [37] after their adsorption to diopside, despite their drastically different chemical structure. The recombinant protein BMP-2 had a somewhat prolonged release compared to lysostaphin, vancomycin, and dexamethasone, with significant amounts measured for up to 48 h and detectable amounts for up to 9 days [28]. This might indicate a different binding mechanism for BMP-2 compared to other substances. Importantly, despite being relatively short, the period of lysostaphin release from diopside of 24 h is still substantially longer than the period of lysostaphin elimination from circulation upon systemic administration. Lysostaphin has a terminal half-life of less than an hour in mice [38] and 1.5 h in rats [31]. Relatively fast elimination was observed in clinical studies of other antibacterial enzymes [39].

Loading of another antimicrobial agent, glycopeptide antibiotic vancomycin, on diopside particles has been described before [36,40]. Unfortunately, the authors did not test the antibacterial activity of the resulting diopside. In our work, diopside loaded with lysostaphin displayed strong antibacterial activity in vitro. This activity, however, was lower than the activity of an equivalent amount of free lysostaphin, which was evident from the higher MIC values and higher concentrations required to destroy the biofilms (Figure 4, Table 1). On the one hand, lysostaphin is gradually released from the diopside during the incubation with planktonic bacteria or biofilms, and the maximal concentration of released lysostaphin is only achieved after 24 h (Figure 2). On the other hand, almost 70% of the adsorbed lysostaphin was bound to diopside very strongly or irreversibly and did not dissociate even after prolonged incubation. Both these factors result in the lower activity of lysostaphin adsorbed to the diopside powder compared to the free lysostaphin. Nevertheless, the amount of released lysostaphin was sufficient to eliminate both planktonic and biofilm-dwelling bacteria in vitro. Furthermore, in vivo diopside is subjected to gradual dissolution and cell-mediated degradation [41]. These processes could potentially free the strongly bound lysostaphin fraction, increasing the overall antibacterial activity of the diopside powder. Although the utility of diopside loaded with lysostaphin in vivo remains to be determined, the presented results indicate that it is a potentially promising approach to prevent the multiplication of pathogenic bacteria inadvertently brought in during surgery or to eliminate the infection pre-existing at the site of a bone defect.

Unlike traditional antibiotics, lysostaphin and other antibacterial enzymes can effectively kill both actively growing and dormant bacterial cells, which makes them potentially more effective in combating bacterial biofilms and preventing the development of chronic infections. However, lysostaphin is only active towards *S. aureus* and, to a lesser extent, coagulase-negative staphylococci [13]. Most other antibacterial enzymes possess relatively narrow specificity as well [42,43]. Thus, the loading of diopside with two or more different antibacterial proteins might be required to achieve the desired spectrum of antibacterial activity. 

To sum up, we have demonstrated that the loading of the diopside powder with the antibacterial protein lysostaphin can grant it strong antibacterial activity towards both planktonic cells and biofilms of *S. aureus*, one of the major causes of implant associated infections. This observation broadens the potential applications of diopside as the material of choice for reconstructive bone surgery. Future studies can be directed at the optimization of protein loading conditions and protocols as well as investigating the possibility of loading diopside with two or more antibacterial proteins with different specificities. Since in many cases an implant has to provide mechanical support, which diopside powder itself cannot offer, further studies should also address the possibility of incorporating it into load-bearing materials and the influence of this procedure on the adsorption of recombinant proteins. Finally, testing of the antibacterial activity of diopside loaded with lysostaphin in vivo is required.

## Figures and Tables

**Table 1:** Antibacterial and anti-biofilm activity of lysostaphin and diopside loaded with lysostaphin.

	Lysostaphin, µg/mL	Diopside, mg/mL (In Brackets—The Respective Amount of Adsorbed Lysostaphin, µg/mL)
MIC	0.10	0.06 (0.31)
MBC	0.10	0.13 (0.68)
CV ^1^	0.02	0.10 (0.52)
MBC-P	0.07	0.20 (1.04)
MBC-B	0.07	0.10 (0.52)

^1^ Concentration resulting in almost-complete biofilm elimination determined by staining of the biofilms with crystal violet.

## Data Availability

Not applicable.
